# Lifetime Planning of Aortic Stenosis Treatment Starts Before the First Valve

**DOI:** 10.1016/j.shj.2026.101085

**Published:** 2026-06-26

**Authors:** Warkaa Shamkhani, Clare Appleby, James Nolan, David J. Cohen, Jorge Chavarria, Mohamed Suleman, Benoy N. Shah, Gilbert H.L. Tang, Mamas A. Mamas

**Affiliations:** a Cardiology Department, Liverpool Heart and Chest Hospital NHS Foundation Trust, Liverpool, UK; b Cardiovascular Research Group, Keele University, Stoke-on-Trent, UK; c Cardiovascular Research Foundation, New York, New York, USA; d Cardiology Department, St. Francis Hospital, Roslyn, New York, USA; e Cardiology Department, San Juan De Dios Hospital, San Jose, Costa Rica; f Cardiology Department, Hamilton Health Sciences, McMaster University, Hamilton, Canada; g Wessex Cardiac Centre, Southampton General Hospital, Southampton, UK; h Department of Cardiovascular Surgery, Icahn School of Medicine at Mount Sinai, Mount Sinai Health System, New York, New York, USA

## Abstract

**CT-guided lifetime planning in severe aortic stenosis.** Preprocedural CT should guide not only valve implantation, but the first lifetime treatment decision. In patients with longer expected survival, CT can identify whether TAVR-first is likely to preserve coronary access and future TAVR-in-TAVR feasibility, or whether SAVR-first should be considered when surgical risk is acceptable. Abbreviations: CT, computed tomography; SAVR, surgical aortic valve replacement; TAVR, transcatheter aortic valve replacement.

**Figure:**
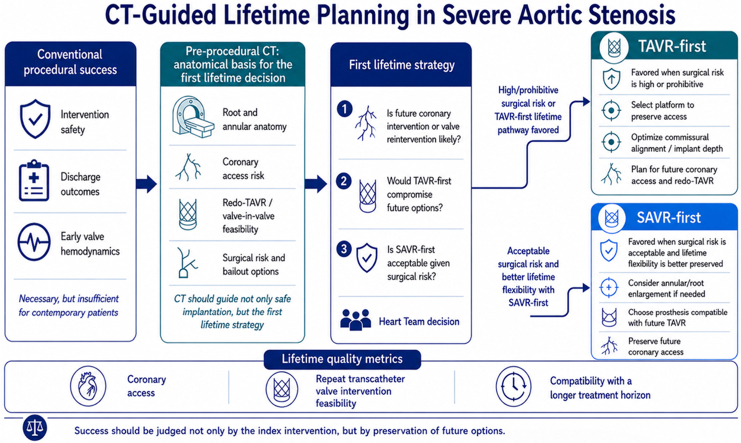

## Introduction: the Illusion of Success

Transcatheter aortic valve (TAV) replacement (TAVR) is no longer viewed as a destination therapy for patients with limited life expectancy. It is increasingly the first valve intervention in patients who may later require coronary access, repeat valve intervention, or surgical bailout. Yet procedural success continues to be judged mainly by short-term safety, outcomes, and hemodynamics. For patients with longer expected survival, the key question is not only whether the valve can be implanted safely, but whether the first-valve strategy preserves future treatment options.^1^

Recent work has emphasized that redo-TAVR feasibility begins with optimization of the index procedure.^2^ We extend this principle upstream: preprocedural computed tomography (CT) should inform not only how TAVR is performed, but whether a TAVR-first approach is the most appropriate first lifetime treatment choice. This opinion piece proposes a practical conceptual framework rather than a validated clinical algorithm to support Heart Team decisions between TAVR-first, surgical aortic valve replacement (SAVR)-first, or TAVR-first strategies with explicit lifetime risk mitigation.

## Trials Established Early Success, Not Lifetime Strategy

Randomized trials established TAVR as an effective alternative to surgery across the surgical-risk spectrum, with favorable early survival, stroke, and hemodynamic outcomes.^3^^,^^4^ However, these trials were not designed to determine whether the index intervention preserves coronary access, enables repeat transcatheter treatment, or supports a lifetime valve management strategy. These questions have become more important as TAVR expands into younger, lower-risk patients with longer expected survival.

Low- and intermediate-risk trials enrolled anatomically selected populations, had limited follow-up, underrepresented younger patients, and did not systematically assess endpoints central to lifetime management, such as coronary access, repeat intervention feasibility, and long-term structural compatibility.^5^ Accordingly, early trial success should not be assumed to define the lifetime suitability of the index strategy.

Longer-term follow-up is beginning to expose signals that early trials were not designed to capture. In Evolut Low Risk, 5-year outcomes remained similar between TAVR and surgery for death or disabling stroke (15.5% vs. 16.4%; *p* = 0.47) and valve performance remained excellent.^5^ Although death or disabling stroke still did not differ significantly by 6 years (23.3% vs. 20.4%; *p* = 0.43), reintervention was higher after TAVR at 6 years (5.5% vs. 3.3%) and in available 7-year follow-up (9.8% vs. 6.0%; subdistribution hazard ratio 1.68; *p* = 0.02), driven mainly by aortic regurgitation.^6^ These findings do not undermine the value of TAVR, but they show why early noninferiority cannot be treated as proof of lifetime durability. The next challenge is to link trial-level outcomes with the anatomical and procedural features of the index intervention that may influence reintervention over time.

## CT as Strategic Gatekeeper, Not Just a Procedural Tool

**Figure 1:**
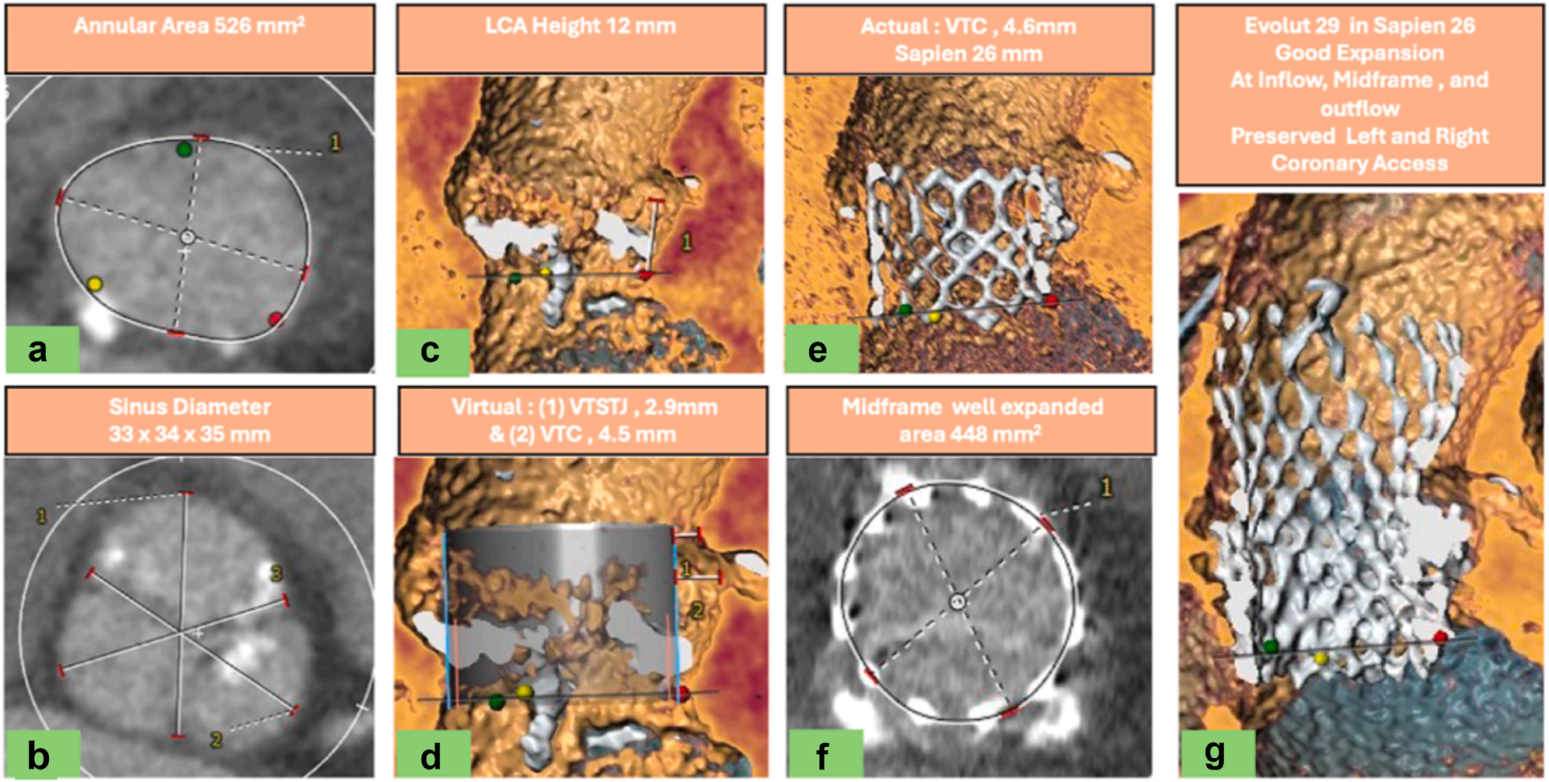
**CT-basedassessment ofcoronary andsinotubularjunctionclearance forredo-TAVRfeasibility.** Preprocedural CT demonstrated **(a)** an annular area of 526 mm^2^, **(b)** sinus of Valsalva dimensions of 33 × 34 × 35 mm, and **(c)** left coronary height of 12 mm. **(d)** Virtual modeling of a 26-mm balloon-expandable valve showed predicted preservation of the VTSTJ (2.9 mm) and VTC (4.5 mm). Postprocedural CT confirmed **(e)** an actual VTC of 4.6 mm after 26-mm Sapien implantation and **(f)** good post-TAVI midframe expansion area (448 mm^2^). **(g)** Redo-TAVR with a 29-mm self-expanding valve within the Sapien valve resulted in good expansion at the inflow, midframe, and outflow levels, with preserved bilateral coronary access and no coronary occlusion or sinus sequestration. Abbreviations: CT, computed tomography; LCA: left coronary height; TAVI, transcatheter aortic valve implantation; TAVR, transcatheter aortic valve replacement; VTC, valve-to-coronary distance; VTSTJ, valve-to-sinotubular junction distance.

**Figure 2:**
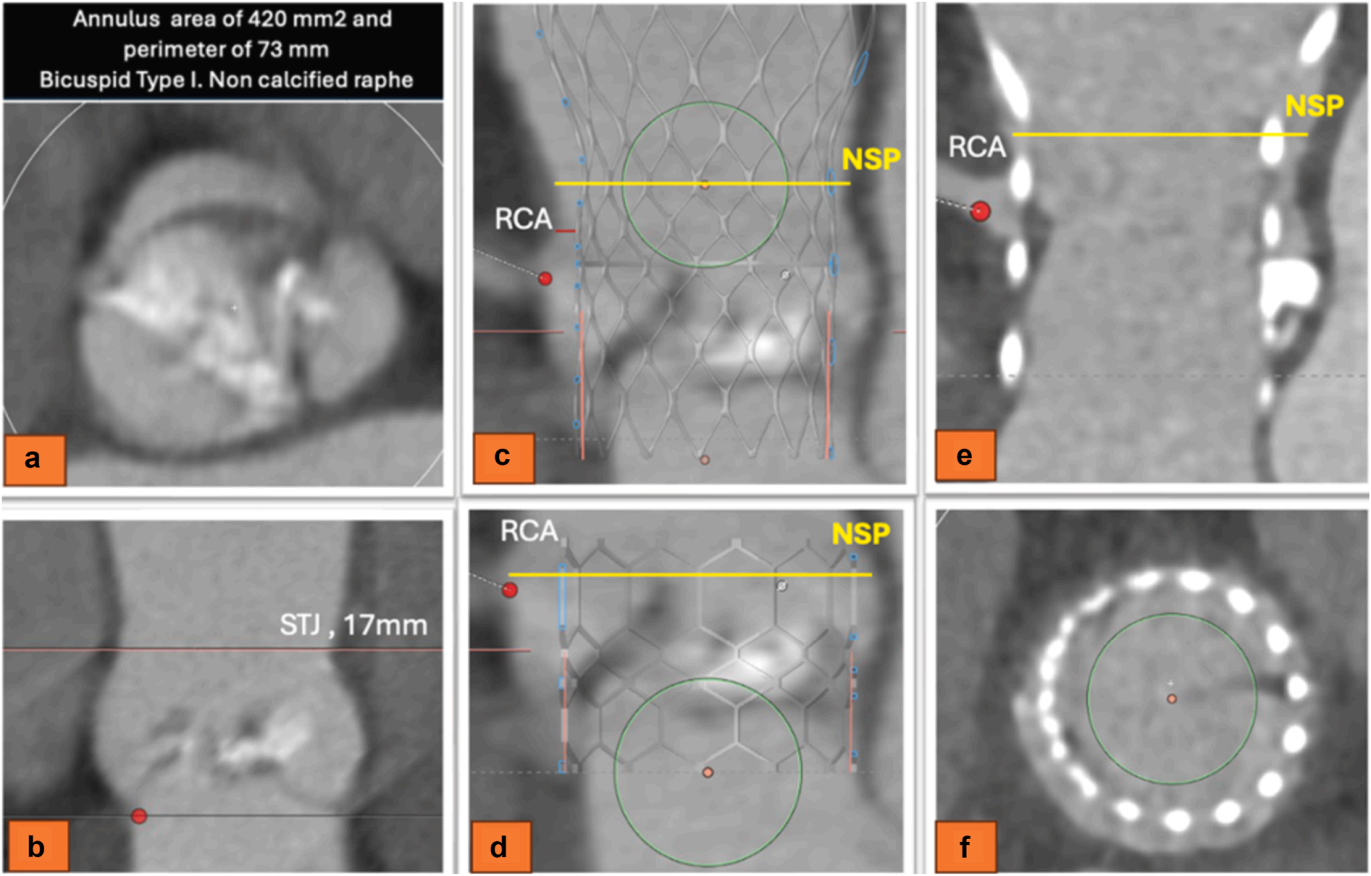
**CT-basedassessment ofcoronaryobstructionrisk in TAVR-in-TAVR.** A 68-year-old patient with bicuspid aortic valve disease previously treated with a 26-mm self-expanding valve (Evolut, Medtronic) developed severe aortic regurgitation due to leaflet prolapse and underwent redo-TAVR with a balloon-expandable valve, coronary protection, and RCA chimney stenting. Baseline CT showed bicuspid valve anatomy with **(a)** noncalcified raphe and asymmetric leaflet calcification, **(b)** STJ height, and **(c)** virtual simulation demonstrating the NSP above the coronary ostia with a self- expanding valve, **(d)** but a lower-risk configuration below the RCA ostium with a balloon-expandable valve and 80/20 deployment profile. Follow-up CT showed the implanted Evolut valve with the RCA origin below the NSP **(e)** and short-axis view of the sinuses **(f)**. The valve-to-sinotubular junction distance (red line) was <2 mm in both sinuses Abbreviations: CT, computed tomography; NSP, neoskirt plane; RCA, right coronary height; STJ, sinotubular junction; TAVR, transcatheter aortic valve replacement.

Beyond annular sizing, preprocedural CT can identify anatomy in which future coronary access and redo-TAVR feasibility may be preserved or compromised (Figure 1). Yet in many Heart Team discussions, CT guides how TAVR is performed but not whether it should be the first intervention for patients with a long treatment horizon. Low coronary height, small sinuses, narrow sinotubular junction, bulky leaflet calcification, unfavorable predicted valve-to-coronary or valve-to-sinotubular junction relationships, and risk of severe prosthesis-patient mismatch should therefore prompt explicit lifetime discussion before selecting a TAVR platform (Figure 2).

In such patients, the key question is whether the first intervention will preserve future coronary access, redo-TAVR feasibility, and bailout options. Importantly, a surgery-first strategy is not a universal “escape hatch” either: CT studies have shown that SAVR in small aortic roots without enlargement may significantly reduce coronary height and leave residual high-risk anatomy for future valve-in-valve TAVR and coronary access after TAVR, particularly when the surgical prosthesis, coronary ostia, and residual root geometry create limited clearance.

When CT suggests that future TAV-in-TAV remains feasible, a TAVR-first strategy may be appropriate if the index procedure is planned to preserve future reintervention. When CT predicts high risk of sinus sequestration, coronary obstruction, or loss of coronary access after redo-TAVR, a SAVR-first strategy should be considered if surgical risk is acceptable. In high or prohibitive surgical risk, a TAVR-first strategy may remain appropriate, but lifetime constraints should be recognized, mitigated where possible, and documented.^7^ These considerations are summarized within a structured lifetime planning framework (Supplementary Table 1).

## Valve Platform, Implant Depth, and Commissural Orientation Should be Considered Lifetime Variables

**Figure 3:**
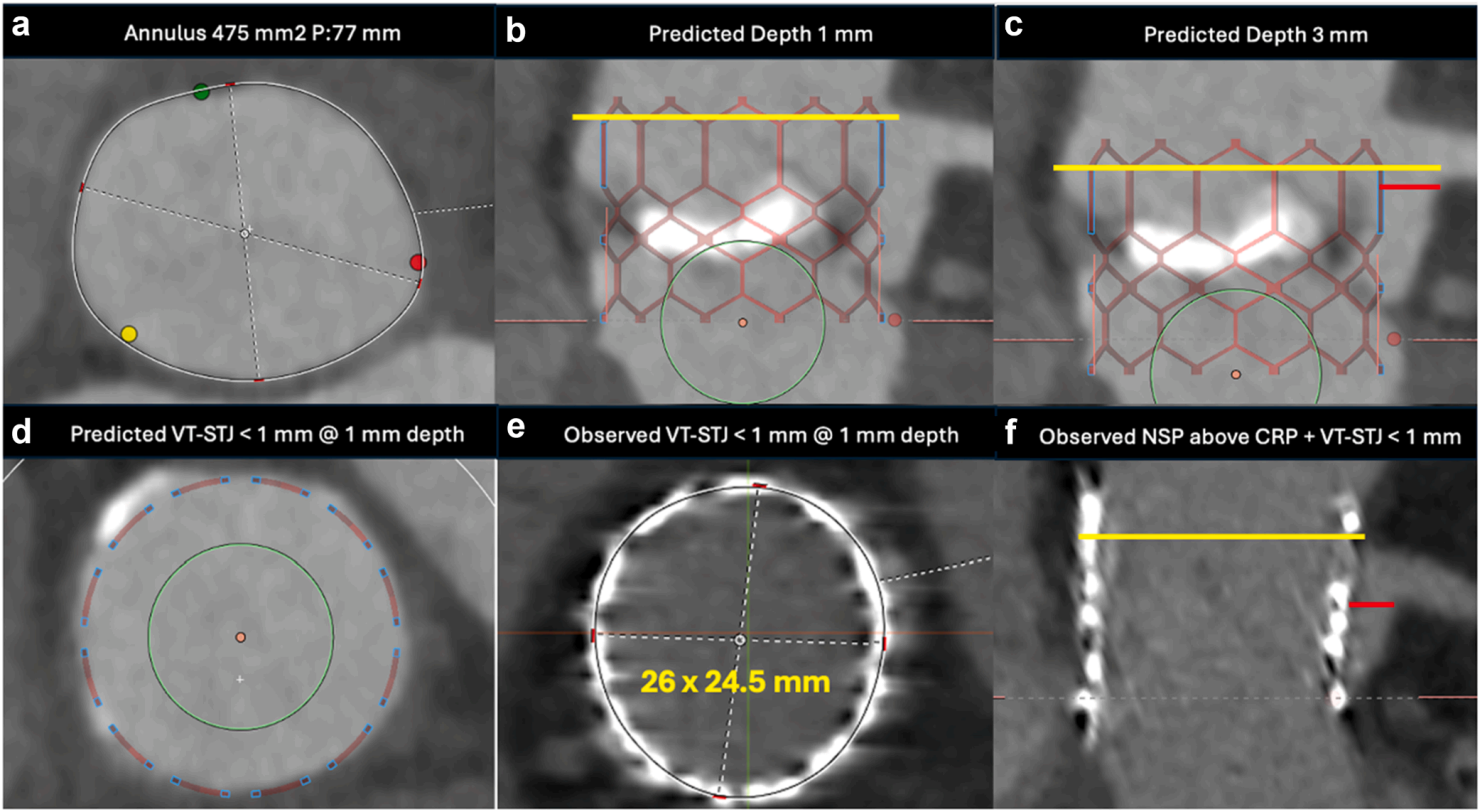
**Impact of initial implementation depth on redo-TAVR feasibility: 26mm valve.** Representative case illustrating how index implantation depth influences the CRZ and redo-TAVR risk. **(a)** Baseline computed tomography demonstrating an annular area of 475 mm^2^. **(b)** Predicted deployment at 1 mm implantation depth positions the NSP above the CRP, creating a high-risk CRZ with minimal VTA <1 mm due to VT-STJ <1 mm. **(c)** Predicted deployment at 3 mm implantation depth lowers the NSP below the sinotubular junction so that the relevant CRZ clearance parameter becomes the VTC, which remains 4.5 mm. **(d and e)** Predicted and observed VT-STJ <1 mm at 1 mm implantation depth confirms near-complete sinotubular junction effacement. **(f)** Postprocedural imaging at 1 mm implantation depth demonstrates persistent high-risk CRZ clearance with VTA <1 mm due to VT-STJ <1 mm. This figure emphasizes that redo-TAVR feasibility is governed by the smallest VTA within the CRZ, rather than by VTC alone. The yellow line denotes the neoskirt plane, and the red line denotes the coronary risk plane. Abbreviation: CRP, coronary risk plane; CRZ, coronary risk zone; NSP, neoskirt plane; TAVR, transcatheter aortic valve replacement; VTA, valve-to-aorta distance; VTC, valve-to-coronary distance; VT-STJ, valve-to-sinotubular junction distance.

Future coronary access and redo-TAVR feasibility are shaped not only by baseline anatomy but also by index procedural choices, including valve platform, implant depth, and commissural orientation (Figures 2 and 3). Thus, even a technically successful implant may preserve or compromise the next intervention.

Platform-specific tradeoffs should be considered explicitly. Supraannular and intraannular taller-frame valves may provide favorable hemodynamics, particularly in small annuli, but may increase the complexity of future coronary access or TAV-in-TAV. Shorter-frame intraannular valves may preserve access in selected anatomies but involve different tradeoffs in gradients and severe prosthesis-patient mismatch. The aim is not to define a universally superior platform but to select the first valve that best matches the patient’s anatomy and lifetime management.

CT simulation studies show that redo-TAVR feasibility is critically determined by the achieved index implant result. SAPIEN 3 (Edwards Lifesciences, Irvine, California), Navitor (Abbott Cardiovascular, Santa Clara, California), and Evolut (Medtronic, Minneapolis, Minnesota) simulations demonstrate that implant depth, commissural alignment, and redo-valve position can determine predicted coronary access and sinus sequestration risk.^8^, ^9^, ^10^ These data support a broader lifetime-planning principle: the first treatment decision should account for the feasibility of a second intervention. Accordingly, implant depth and commissural alignment should be treated as lifetime-management variables, not just procedural endpoints.

## Frame Deformation and Expansion Predict Valve Durability

**Figure 4:**
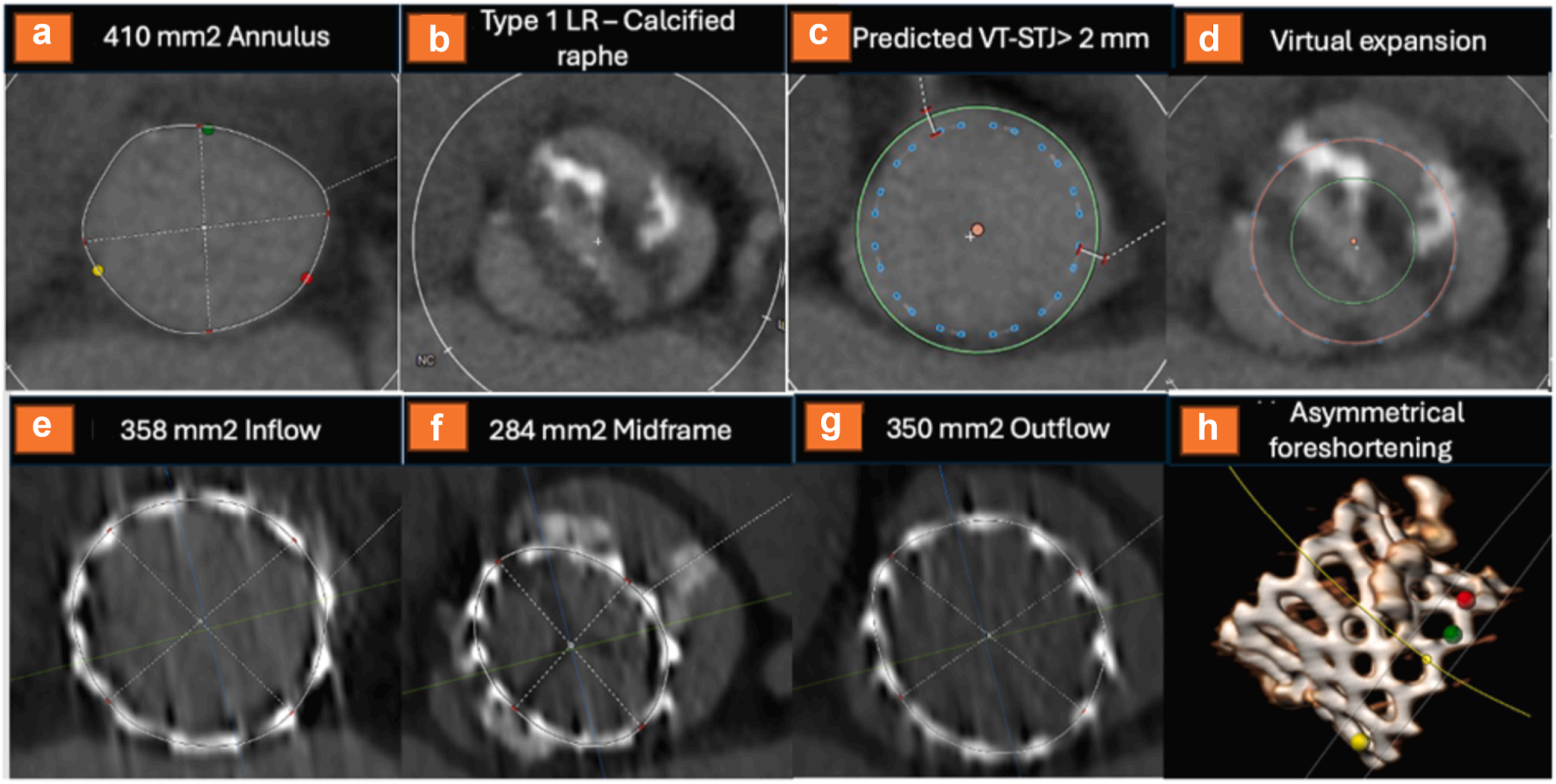
**CT-based mechanistic assessment of late structural valve deterioration after TAVR in bicuspid anatomy.** A 62-year-old woman with severe aortic stenosis and acute decompensated heart failure (left ventricular ejection fraction, 20%) underwent urgent TAVR, with recovery of ejection fraction to 56%. At 5-year follow-up, she developed VARC-3 structural valve deterioration with elevated transvalvular gradients and severe patient-prosthesis mismatch. Preprocedural CT showed **(a)** an annular area of 410 mm^2^, **(b)** bicuspid aortic valve (Sievers type 1, left-right fusion) with calcified raphe, **(c)** predicted valve-to-sinotubular junction distance >2 mm, and **(d)** software-derived virtual expansion of a nominal 23-mm balloon-expandable valve (Sapien platform). In the context of heavy raphe calcification, this anatomy suggested a likely constraint to full frame expansion. Follow-up CT showed **(e)** preserved inflow expansion (358 mm^2^), **(f)** severe midframe underexpansion (284 mm^2^) with marked eccentricity, **(g)** preserved outflow expansion (350 mm^2^), and **(h)** volume-rendered reconstruction demonstrating asymmetric foreshortening and marked frame underexpansion. These findings provide a mechanistic explanation for late structural valve deterioration and limit the feasibility of redo transcatheter valve-in- valve intervention. She was referred for elective surgical explantation and surgical bioprosthetic valve replacement Abbreviation: CT, computed tomography; LR, left and right coronary cusps; TAVR, transcatheter aortic valve replacement; VARC, Valve Academic Research Consortium; VT-STJ, valve-to-sinotubular junction distance.

Frame geometry may also influence lifetime compatibility. In heavily calcified anatomy, particularly bicuspid valves with bulky raphe or leaflet calcification, the index valve may expand asymmetrically or adopt a distorted final geometry. This may affect leaflet function, durability, coronary access, and future transcatheter reintervention feasibility (Figure 4).

When preprocedural CT suggests a high likelihood of constrained expansion, this should inform valve selection, deployment strategy, and whether a TAVR-first strategy remains the most suitable lifetime option.

## A Lifetime-Oriented Approach to Decision-Making

We propose that lifetime-oriented aortic valve replacement planning should begin with three practical questions (Graphical Abstract). First, is the patient’s expected clinical course long enough that future coronary intervention, valve reintervention, or both are reasonably likely? Second, does preprocedural CT suggest that TAVR-first would preserve coronary access and future TAVR-in-TAVR feasibility? Third, if TAVR-first appears anatomically unfavorable, does surgical risk support SAVR-first, or is TAVR-first still the safest initial treatment despite recognized lifetime constraints? A more detailed pathway incorporating frailty, age, CT anatomy, surgical risk, TAVR-specific risk, and Heart Team documentation is shown in Supplementary Figure 1.

In this framework, TAVR-first is most compelling when anatomy allows safe implantation while preserving future coronary access and repeat transcatheter treatment options. SAVR-first becomes more attractive when CT predicts that TAVR-first would create a high-risk lifetime pathway and surgical risk is acceptable. In high or prohibitive surgical risk, TAVR-first may remain appropriate, but expected future limitations should be recognized, mitigated where possible, and documented.

This approach does not replace multidisciplinary judgment; it makes that judgment more explicit. For example, a younger patient with low surgical risk but unfavorable CT anatomy for coronary access or redo-TAVR may be better served by a SAVR-first strategy, whereas a higher-risk patient with favorable anatomy may undergo TAVR first with procedural optimization to preserve future options.

## Limitations and Future Directions

This framework is pragmatic but is based largely on CT-derived anatomy, simulation studies, and mechanistic reasoning rather than prospective lifetime outcome data. Not every predicted limitation in coronary access or redo-TAVR feasibility will become clinically relevant, and some future constraints may not be apparent from baseline CT alone.

Preprocedural CT should therefore be considered the anatomical starting point, not the final determinant of lifetime feasibility. The achieved implant result, including valve depth, commissural orientation, frame expansion, postdilatation, and interaction with the sinotubular junction, may materially alter future options. Leaflet modification techniques may improve redo-TAVR feasibility in selected patients but should not be viewed as a substitute for careful index planning.

Prospective studies are needed to link baseline CT anatomy, achieved implant geometry, coronary reaccess, redo-TAVR feasibility, surgical bailout, valve performance, and long-term clinical outcomes. Artificial intelligence–enabled tools may improve the reproducibility of CT-based lifetime planning, but the clinical framework must come first.

## Conclusion: Reframing the Question to Lifetime Management

In younger patients and those with longer expected survival, TAVR success should no longer be judged solely by index procedural safety, but by whether the first-valve strategy preserves future treatment options. Preprocedural CT can identify anatomy in which coronary access, redo-TAVR feasibility, or both may later become constrained. The challenge is therefore not only to implant the first valve successfully, but to choose and perform the first intervention in a way that remains compatible with the patient’s likely lifetime pathway. In contemporary lifetime-oriented TAVR planning, management should begin before the first valve is implanted.

## Ethics Statement

The use of fully deidentified patient CT images included in this opinion piece was reviewed and approved, or deemed exempt from formal ethical review, by the relevant institutional ethics committees at McMaster University/Hamilton Health Sciences, Canada, and San Juan de Dios Hospital, Costa Rica. No identifiable patient information is included.

## Funding

The authors have no funding to report.

## Disclosure Statement

Gilbert H.L. Tang has received speaker's honoraria and served as a physician proctor, consultant, advisory board member, TAVR publications committee member, RESTORE study steering and screening committee member, APOLLO trial screening committee member, and IMPACT MR steering committee member for Medtronic; has received speaker's honoraria and served as a physician proctor, consultant, advisory board member, ENVISION trial screening committee member, and TRILUMINATE trial anatomic eligibility and publications committee member for Abbott Structural Heart; and has served as an advisory board member for Boston Scientific, Anteris, Pi-Cardia, Philips, Edwards Lifesciences, Peija Medical, and Shenqi Medical Technology.

The other authors have no conflicts to declare.
