# Supplementary material for: Lifetime Planning of Aortic Stenosis Treatment Starts Before the First Valve
Source: Struct Heart. 2026 Jun 26;10(9):101085. doi: 10.1016/j.shj.2026.101085 (PMC13482539; doi:10.1016/j.shj.2026.101085)
Supplement: Supplementary file 1 [file mmc1.docx]

**Supplementary Figure 1**

| **Supplementary Table 1. CT-Guided Framework for Lifetime TAVR Planning** | | | | |
| --- | --- | --- | --- | --- |
| **Domain** | **Favorable lifetime profile** | **Higher-risk lifetime profile** | **Why it matters** | **Strategic implication** |
| **Expected clinical course** | Younger age, longer expected survival, good functional reserve, acceptable surgical risk | Severe frailty, limited life expectancy, prohibitive comorbidity burden | Longer-survival patients are more likely to need future coronary access or valve reintervention | In longer expected survival patients, judge success by preservation of future options, not only early procedural safety |
| **CT anatomy** | Adequate coronary height, large sinuses, wide STJ, favorable VTC and VT-STJ | Low coronaries, small sinuses, narrow STJ, bulky leaflet calcium, unfavorable VTC or VT-STJ | Anatomy may determine whether redo-TAVR and coronary re-access remain feasible | Use CT as a strategic gatekeeper before choosing TAVR-first versus SAVR-first |
| **Coronary access** | Predicted coronary access remains feasible after index TAVR and redo-TAVR | Predicted difficult coronary access, coronary obstruction, or sinus sequestration | Loss of coronary access may limit future PCI or emergency bailout | Select valve platform, implant depth, and commissural orientation to preserve access |
| **Valve platform** | Platform matches anatomy and likely future pathway | Platform risks compromising coronary access or redo feasibility in constrained anatomy | Different frame heights, leaflet positions, and cell geometry create different lifetime trade-offs | Choose the first valve as part of a lifetime strategy, not only for index hemodynamics |
| **Implant depth and commissural orientation** | Planned depth and alignment preserve future coronary access and redo feasibility | High/shallow implant or unfavorable alignment risks future access limitation | A technically successful implant can still create long-term constraints | Treat implant depth and alignment as lifetime-management variables |
| **Frame expansion and root interaction** | Symmetric expansion with limited eccentricity or distortion | Heavy calcium, bicuspid raphe, asymmetric underexpansion, distorted frame geometry | Distortion may affect leaflet function, durability, coronary access, and redo-TAVR feasibility | Incorporate calcium burden and predicted expansion into valve choice and deployment strategy |
| **SAVR-first pathway** | Acceptable surgical risk and surgical strategy that preserves future ViV options | High surgical risk, small surgical valve, reduced coronary height, constrained residual root | Surgery-first is not automatically future-proof | If SAVR is chosen, plan prosthesis size and root strategy with future ViV-TAVR in mind |
| **Heart Team decision** | Clear documentation of TAVR-first, SAVR-first, or TAVR-first with mitigation | Decision based mainly on index procedural feasibility | The first intervention may determine the next 10–20 years of options | Document the expected lifetime pathway and anticipated future limitations |
| This table summarizes a practical framework for integrating patient clinical horizon, CT anatomy, coronary access, valve platform, implant strategy, frame geometry, surgical risk, and Heart Team documentation into the first lifetime treatment decision. The framework is intended to support structured discussion of TAVR-first, SAVR-first, or TAVR-first with explicit mitigation, rather than to function as a validated clinical algorithm. | | | | |
